# Engineering Next-Generation CAR-T Cells for Better Toxicity Management

**DOI:** 10.3390/ijms21228620

**Published:** 2020-11-16

**Authors:** Alain E. Andrea, Andrada Chiron, Stéphanie Bessoles, Salima Hacein-Bey-Abina

**Affiliations:** 1 Laboratoire de Biochimie et Thérapies Moléculaires, Faculté de Pharmacie, Université Saint Joseph de Beyrouth, Beirut 1100, Lebanon; alain.andrea@net.usj.edu.lb; 2 Université de Paris, CNRS, INSERM, UTCBS, Unité des Technologies Chimiques et Biologiques pour la Santé, F-75006 Paris, France; andrada.chiron@parisdescartes.fr (A.C.); stephanie.bessoles@parisdescartes.fr (S.B.); 3 Clinical Immunology Laboratory, Groupe Hospitalier Universitaire Paris-Sud, Hôpital Kremlin-Bicêtre, Assistance Publique-Hôpitaux de Paris, 94275 Le-Kremlin-Bicêtre, France

**Keywords:** chimeric antigen receptor, CAR-T cell, engineering, cancer, immunotherapy, toxicity, cytokine release syndrome

## Abstract

Immunoadoptive therapy with genetically modified T lymphocytes expressing chimeric antigen receptors (CARs) has revolutionized the treatment of patients with hematologic cancers. Although clinical outcomes in B-cell malignancies are impressive, researchers are seeking to enhance the activity, persistence, and also safety of CAR-T cell therapy—notably with a view to mitigating potentially serious or even life-threatening adverse events like on-target/off-tumor toxicity and (in particular) cytokine release syndrome. A variety of safety strategies have been developed by replacing or adding various components (such as OFF- and ON-switch CARs) or by combining multi-antigen-targeting OR-, AND- and NOT-gate CAR-T cells. This research has laid the foundations for a whole new generation of therapeutic CAR-T cells. Here, we review the most promising CAR-T cell safety strategies and the corresponding preclinical and clinical studies.

## 1. Introduction

For many decades, cancer therapy has relied primarily on surgery, chemotherapy, radiotherapy, and bone marrow transplantation (BMT). Recently, advances in immune response stimulation have yielded lasting remissions and have established immunotherapy as the fifth pillar of cancer therapy. In 1891, William Coley was the first physician to attempt to exploit the immune system for cancer treatment [1]. In the 1950s, the success of BMT laid the foundations for the development of adoptive cell therapy, with the infusion of cells into patients with hematological cancers proving that T cells have the power to kill cancer cells. Another key milestone was the discovery of monoclonal antibodies (mAbs) in 1975 by Georges Köhler and Cesar Milstein (the winners of the Nobel Prize in Medicine in 1984) [2]. These mAbs served as research tools before being used therapeutically. The emergence of mAbs as anti-cancer drugs was facilitated by the ability to mass-produce them in the laboratory, which led to the approval of rituximab for lymphoma treatment in 1997 [3]. In 1993, Zelig Eshhar and colleagues designed the first chimeric antigen receptor (CAR). This chimera contained the T-cell receptor (TCR)’s constant domains and the antibody’s variable domain, thus combining the latter’s high specificity and affinity with the T lymphocytes’ functional capacities (i.e., homing, tissue penetration, and cytotoxicity). The novelty of this new receptor relied on the ability to direct the specificity of genetically modified T cells towards a chosen tumor antigen in an MHC-independent context [4].

**Figure 1:**
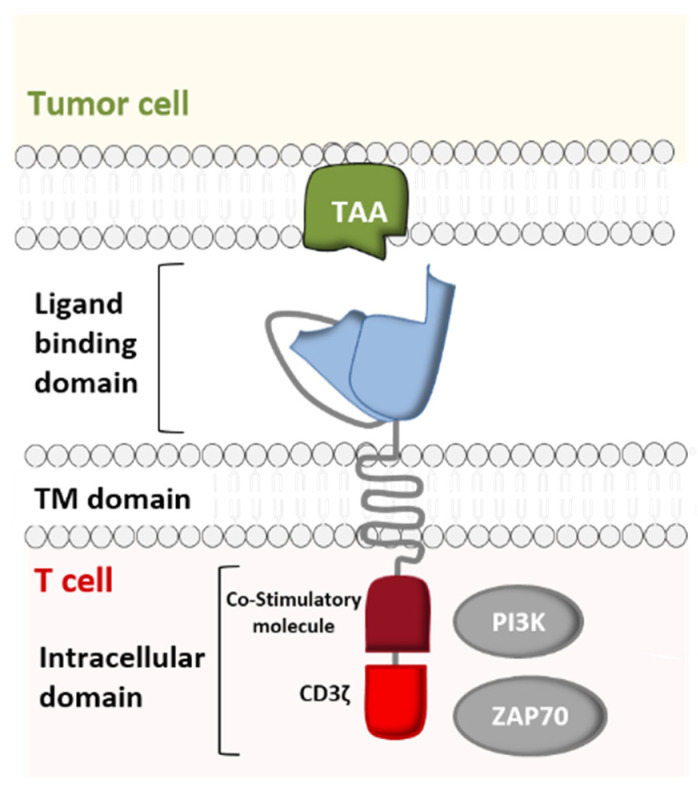
Structure of a chimeric antigen receptor (CAR)—a cell-surface receptor comprising an extracellular ligand binding domain (which recognizes a specific tumor-associated antigen (TAA)), a transmembrane (TM) domain, and an intracellular signaling domain. The latter consists of a T cell activation domain and one or more co-stimulatory domains (CD27, CD28, ICOS, 4-1BB, and/or OX40) (Adapted from Lee and Kim, 2019).

Just like immune checkpoint inhibitors [5], CAR-T cell immunotherapy is the culmination of more than 60 years of in-depth research on the immune system, genetic engineering, antibody therapy, and on the oncogenic mechanisms in hematological cancers. The development of CARs resulted from extensive research by several researchers, including not only Steven Rosenberg, Carl June, and Michel Sadelain but also Bryan Irvin and Arthur Weiss, who demonstrated that TCR-independent activation of T cells was possible. In order to study the role of the CD3 ζ chain in TCR-mediated signal transduction, these researchers constructed a chimeric protein linking CD8’s extramembrane domain and transmembrane domain (TM) to the cytoplasmic domain of the ζ chain. They noted that when expressed on a T-cell, the CD8/ζ pair is able to transduce signals (following the interaction of a ligand with the CD8 extracellular domain (ECD)) in the same way as the physiological TCR/CD3 pair [6,7] (Figure 1).

Furthermore, various studies determined the role of individual immunoreceptor tyrosine-based activation motifs (ITAMs) in the modulation of TCR/CD3 complex signaling. Experiments with T cells indicated that the cytoplasmic domains of the CD3ε and CD3ζ subunits were independently capable of providing activation signals similar to those of the intact TCR [6,8,9]. Hence, Eshhar et al. came up with the idea of reinforcing the CD8/ζ receptor’s activity by replacing the CD8 ECD with the variable fragments of a single-chain antibody (scFv) directed against a specific antigen (Figure 1). This allowed CAR-T cells to acquire a third important property: a broad target range. In contrast to the TCR, antibodies are capable of recognizing carbohydrate and lipid antigens as well as peptide antigens [10]. Moreover, the fact that antibodies (unlike the TCR) are able to bind to tumor antigens without MHC-restricted presentation allows CAR-T cells to attack tumor cells that may have down- regulated their MHC expression as an immune escape mechanism [11,12,13]. Following the advent of first-generation CARs, second- and third-generation CARs with greater stimulatory activity were created by engineering the intracellular co-stimulatory domains [14].

The generation of specific CARs (whether patient-derived (autologous) or donor-derived (allogenic)) starts with T cell collection by leukapheresis and is followed by ex vivo re-engineering and expansion of the T cells (so they express specific CARs), and then reinfusion into the patient. Upon recognition of the targeted tumor antigen, the CAR-T cells proliferate and specifically kill the cancer cells [15]. Advances in CAR-T cell therapy offer new options for treating refractory malignancies, and clinical trials in indications of various hematologic or solid cancers are ongoing. The clinical success of CD19-targeted CAR-T cell therapy in B-cell acute lymphoblastic leukemia (B-ALL) and the promising data in B-cell non-Hodgkin’s lymphoma (B-NHL) and chronic lymphocytic leukemia (CLL) are driving extensive research in this new field. These spectacular advances have led to marketing authorizations for Yescarta™ (axicabtagene ciloleucel, from Kite Pharma/Gilead) and Kymriah™ (tisagenlecleucel, from Novartis). These two biologics (both of which target CD19) were approved for the treatment of certain hematological cancers by the United States Food and Drug Administration (FDA) in 2017 and by the European Medicines Agency in 2018.

However, the effectiveness of CAR-T cell adoptive transfer is diminished by “on-target/off-tumor” effects and the appearance of adverse reactions. Indeed, potentially fatal cytokine release syndrome (CRS) occurs in up to 80% of patients treated with anti-CD19 CAR-T cells. CRS can lead to widespread organ toxicities, including cardiovascular disorders (sinus tachycardia hypotension, decreased left ventricular ejection fraction, QT prolongation, and arrhythmia), renal disorders (kidney failure, tumor lysis syndrome, elevated serum creatinine, and electrolyte disturbances such as hyponatremia, hypokalemia and hypophosphatemia), hepatic and gastrointestinal disorders (elevated serum transaminases and bilirubin, nausea, vomiting, and diarrhea), immune disorders (with an elevated risk of bacterial, viral and fungal infections), hematologic disorders (grade 3–4 anemia, leukopenia, thrombocytopenia, neutropenia, and lymphopenia, disseminated intravascular coagulation, prolongation of partial thromboplastin time or prothrombin time, and decreased fibrinogen), respiratory disorders (pulmonary edema, hypoxia, dyspnea, and pneumonitis), musculoskeletal disorders (elevated creatine kinase), and neurologic disorders (global encephalopathy, aphasia, tremor, ataxia, hemiparesis, and cranial nerve palsies). Neurological adverse events can occur either during CRS or after its resolution [16]. Therefore, the need to minimize undesirable immune responses is critical, and recent efforts to improve CAR-T cell therapy have focused on both effectiveness and safety [17]. Abbreviations used are summarized in Table 1.

## 2. From Conventional CAR-T Cells to Next-Generation CAR-T Cells

**Figure 2:**
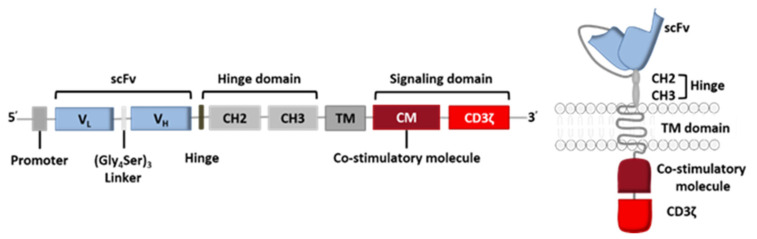
General coding sequence for a CAR.

The CAR construct is based on genetic engineering of the coding DNA sequence, where each set of genes codes for one of the receptor’s five components: the variable fragments of a specific mAb directed against the target antigen (scFv), the hinge region, the TM domain, and the signaling domain (composed of the co-stimulatory CM domain and the CD3ζ domain) (Figure 2).

**Figure 3:**
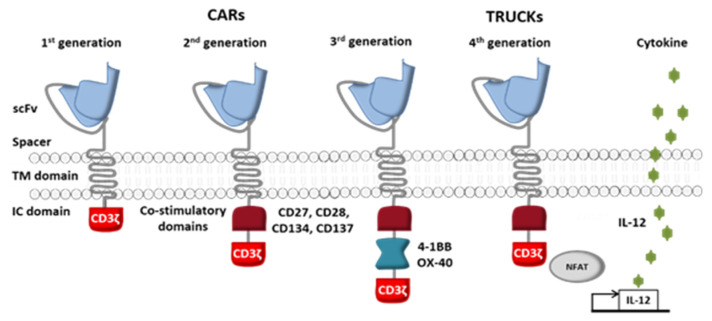
The four generations of CAR-T cells. First-generation CARs were composed of antibody single-chain variable fragments (scFvs) fused through a TM domain to the cytoplasmic tail of the TCR signaling component CD3ζ. Second-generation CARs had improved CAR-T cell proliferation and cytotoxicity, thanks to the addition of co-stimulatory signaling domains (such as CD27, CD28, CD134, and CD137). Third-generation CAR-T cells were generated by adding a second co-stimulatory signaling domain (such as OX-40, also known as CD134, or 4-1BB, also known as CD137) to second-generation constructs. Fourth-generation CAR-T cells, also known as “T cells redirected for universal cytokine-mediated killing” (TRUCKs) were then engineered to secrete transgenic cytokines (like interleukin-12) within the targeted cancer and thus attract more immune cells (such as natural killer (NK) cells and macrophages). (Adapted from Smith et al., 2016).

The receptor’s intracellular domain is the functional domain that enables signal transduction and T cell activation. All clinically tested CARs contain one or two co-stimulation domains (CD28 [18], 4-1BB (CD137) [19,20], OX40 [19], DAP10 [18] and/or CD27 [21]) in addition to the CD3ζ chain. However, other domains have been preclinically tested and have given rise to fourth-generation CAR-T cells (Figure 3) [22]. Given the great heterogeneity of cancer cells in solid tumors, considerable efforts are currently being made to diversify the antitumor response by playing on the recruitment of other immune cells.

The stimulation of a powerful immune response capable of eradicating both hematological cancers and solid tumors with minimal side effects is still a major challenge. There are two major criteria for target specificity: safety, and the persistence of adoptively transferred CAR-T cells. Ideally, CAR-T cells should target only tumor cells and thus spare healthy cells and tissues [23]. Selecting a valid target antigen for CAR-T cells is not easy. Firstly, the ideal target molecule must be a surface tumor antigen that is stably expressed on almost all tumor cells. Furthermore, this molecule should be expressed weakly on healthy tissues, in order to avoid “off-target” side effects associated with the destruction of healthy cells. Expression of the target antigen on vital organs (such as the brain and heart) or hematopoietic stem cells (the “on-target/off-tumor” effect) is redhibitory. For a particular cancer, truly tumor-specific antigens expressed by a large proportion of patients are rare. In contrast to tumor-specific antigens resulting from a gene mutation and expressed exclusively on tumor cells, TAAs are over-expressed on tumor cells but can also be found at low levels on normal cells [24]. A significant example of toxicity associated with the non-exclusive expression of a TAA on tumor cells is illustrated by the case of a patient with metastatic colorectal cancer who received an infusion of autologous CAR-T cells directed against the human epidermal growth factor receptor-2 (HER2)/neu (ErbB2) and who experienced acute respiratory distress and then fatal pulmonary edema [25]. This pulmonary toxicity was likely to be due to the expression of ErbB2 on pulmonary epithelial cells [25]. Thus, a candidate target antigen for CAR-T cells must fulfill stringent criteria: (i) specificity, (ii) the ability to trigger a response that is sufficiently powerful and long-lasting to destroy the target, and (iii) escape from tumor inhibitors, and (iv) limited toxicity. Based on these four criteria, researchers engineering next-generation CAR-T cells have focused on modifying the CAR and other cellular variables.

### 2.1. CAR-T Cells with an OFF-Switch

Several existing or emerging methods for limiting the toxicity of CAR-T cells have been suggested. These include the incorporation of an “OFF-switch” (such as suicide and elimination genes) or a small-molecule-assisted shutoff (SMASh) CAR into genetically modified T cells. Given the expression of one of these genes by CAR-T cells, an external molecule (either a chemical compound or a mAb) could be used as an antidote if CAR-T cells are overactivated.

#### 2.1.1. Suicide Gene Switch

To date, two suicide genes have been integrated into CAR-T cells and tested in the clinic: the gene coding for herpes simplex virus thymidine kinase (HSV-TK) and the gene coding for inducible caspase-9 (iCasp9).

HSV-TK is a conventional method that has been used in the laboratory and in the clinic to induce T cell death (by administration of ganciclovir (GCV)) following the emergence of severe graft-versus-host disease (GvHD) associated with the therapeutic infusion of donor lymphocytes after allogeneic BMT. It is the suicide gene that has been most extensively tested in humans, and is known to be safe and effective [26]. Thus, in order to avoid the overactivation of CAR-T cells, HSV-TK has been incorporated into the CAR. Ganciclovir treatment leads to the formation of a toxic molecule [27,28] and the activation of caspase-8’s apoptotic function, with the induction of ligand-independent CD95 (Fas) aggregation and expression of Fas-associated protein with a death domain [29]. Although this strategy presents several advantages, it also has several limitations: (i) the induction of immunoreactivity against genetically modified cells [30], and (ii) a slow response (up to several days), due to the latency of genome incorporation. In fact, HSV-TK is a cell-cycle-dependent suicide gene, so GCV-based inhibition of HSV-TK-expressing T lymphocytes requires cell proliferation [31]. For these reasons, HSV-TK is not a good candidate for safer CAR-T cell therapy.

**Figure 4:**
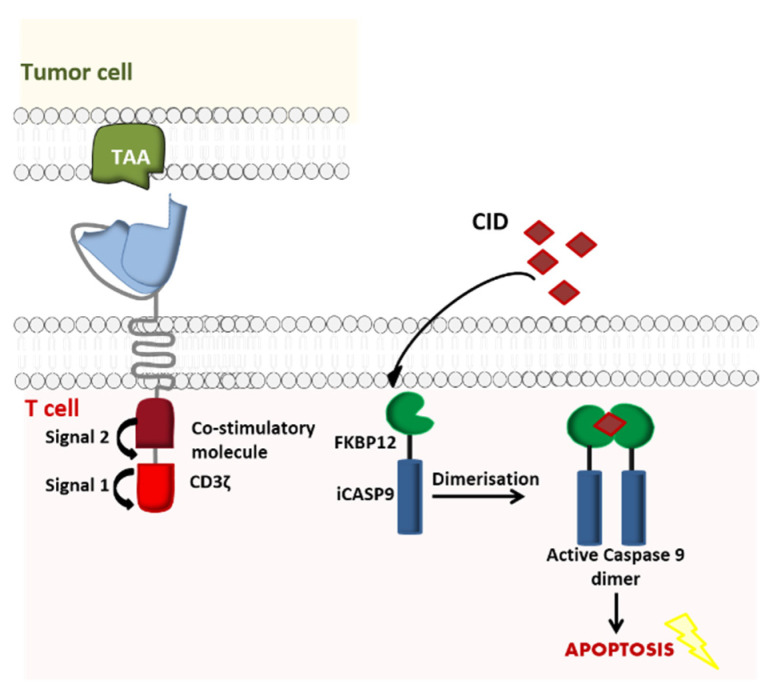
Activation of iCasp9 induces the death of transduced cells. iCasp9 is synthetized in the transduced CAR-T cell as a homodimer. Administration of the chemical inducer of dimerization (CID) AP1903 causes iCasp9 dimerization and thus activation, which in turn triggers a signaling cascade leading to the apoptosis of the CAR-T cells (Adapted from Li and Zhao, 2017).

Similarly, iCasp9 is a safe, effective apoptosis system [32] used initially to prevent GvHD in patients having undergone hematopoietic stem cell transplantation (HSCT). Its use has now been broadened to CAR-T cell therapy. In this novel model, the suicide gene incorporates the intracellular part of the human caspase 9 protein, which is merged to a drug-binding domain comprising of an FK50-binding protein. The latter has a high affinity for a chemical inducer of dimerization (CID, a small molecule called AP1903) that can be intravenously administered in the event of adverse effects (such as life-threatening CRS) following CAR-T cell administration. Once the CID has been injected, it induces cross-linking of iCasp9’s drug-binding domain. In turn, dimerization activates the caspase and induces the apoptosis of the modified CAR-T cells [33] (Figure 4).

In vitro and in vivo experiments show that with a dose of 10 nM AP1903, the iCasp9 suicide gene can kill ~99% of T cells [34]. Lymphocytes that have integrated the iCasp9 gene have certain advantages over those having integrated the HSV-TK suicide gene, such as rapid activation (and therefore rapid cell death) and the humanized nature of the iCasp9 system (reducing the immunogenic risk).

Hoyos et al. were the first to perform a preclinical study of second-generation CD19-targeted CAR-T cells that co-expressed IL-15 and iCasp9. The researchers found that iCasp9-IL15-CD19 CAR-T cells were successfully eliminated within 72 h of AP1903 administration [35]. In another preclinical study of the therapeutic effect of iCasp9-CD20CAR-Δ19 T cells directed against multiple CD20+ tumor cell lines, mice were injected with the CD20+ Raji tumor cell line with firefly luciferase expression in order to noninvasively quantify tumor progression over time. By the end of the 90-day observation period, 69% of the CAR-T cell-treated mice were still alive and showed no firefly luciferase bioluminescence. The long-term complete remission rate was 68% in the cell-treated group and 0% in the control group. Moreover, 90% of the CAR-T cells were eradicated 24 h after the administration of 20 nM AP1903, and 98% were eradicated 72 h after [36]. Many other preclinical studies have also shown promising results for the iCasp9 safety switch in B cell lymphoid malignancies with iCasp9-CD20CAR-ΔNGFR T cells [37], in acute myelogenous leukemia (AML) with iCasp9-CD33CAR-Δ19 cells [38], and in chronic myeloid leukemia (CML) with iCasp9-IL1RAPCAR-Δ19 cells [39] (Table 2).

Based on these encouraging results, Phase I efficacy and safety trials of the iCasp9/AP1903 CAR-T technology have been initiated in several indications: diffuse intrinsic pontine glioma (DIPG), spinal diffuse midline glioma (DMG), relapsed/refractory neuroblastoma, refractory/metastatic sarcoma, relapsed/refractory acute lymphoblastic leukemia (ALL), relapsed/refractory B-cell lymphoma, and other solid cancers (Table 3).

While waiting for the results of these clinical trials to be published, it would be useful to better understand the positive clinical results obtained with iCasp9 as a safety switch strategy for patients undergoing allogeneic HSCT. In a Phase I clinical study (NCT00710892), five pediatric patients (aged between 3 and 17 years) with relapsed acute leukemia underwent allogeneic HSCT. Four of the five patients developed cutaneous GvHD within 14 to 42 days of the first T lymphocyte infusion. They were treated with a single dose of the CID AP1903. This eradicated 90% of the genetically modified T cells within 30 min. Even though this example is not related to CAR-T cell therapy, it illustrates the power of the iCasp9 tool in the treatment of GvHD and prevention of its recurrence [34]. Many other similar clinical trials (e.g., ACTRN12614000290695, NCT01494103) have also showed good efficacy, which makes us optimistic about the clinical prospects for iCasp9 CAR-T cells.

#### 2.1.2. Elimination Markers

In the event of uncontrolled toxicity, genetically engineered CAR-T cells can also be eliminated by using clinically approved mAbs to target a co-expressed, truncated cell-surface protein. The underlying elimination mechanisms in CAR-T cell depletion are antibody-dependent cellular cytotoxicity (ADCC) and complement-dependent cytotoxicity. The truncated protein may be a cluster of differentiation (CD)-type polypeptide or a small epitope peptide recognized by a mAb [23]. The two commonly used truncated proteins are CD20 (recognized by the anti-CD20 mAb rituximab) [40,41], and the truncated human epidermal growth factor receptor polypeptide (huEGFRt, recognized by the anti-EGFR mAb cetuximab) [42,43]. Along with the CD20-rituximab and huEGFRt-cetuximab elimination switches, Kieback et al. suggested introducing a 10 amino-acid tag derived from the c-Myc protein into the recombinant antigen receptor, to enable in vivo elimination of the engineered T cells by administration of an anti-Myc mAb [44]. Despite the preclinical effectiveness of this strategy, the absence of a clinically approved antibody specific for c-Myc makes it ineligible for clinical trials.

**Figure 5:**
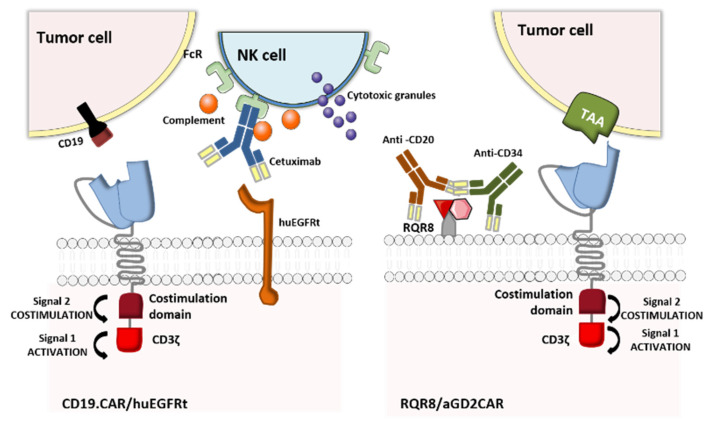
CAR T cells with an elimination marker. huEGFRt and CD20 have been tested as elimination marker systems co-delivered with CAR-T cells. The huEGFRt suicide molecule can be activated with an anti-EGFR cetuximab. In contrast, RQR8 (a 136-amino-acid marker with epitopes from CD34 and CD20) has a dual role, by acting simultaneously as a selection marker when CD34 is targeted with an anti-CD34 MAb (QBEnd/10) and as a suicide molecule when CD20 is targeted with rituximab (Adapted from Li and Zhao, 2017).

Retroviral transfer of human CD20 as an elimination marker for CAR-T cell therapy has proven to be efficacious in many preclinical studies: rituximab ensured the depletion of 86% to 97% of CD20-expressing lymphocytes [40,45,46]. Furthermore, the binding between CD20 and CD34 in a highly compact epitope (RQR8, developed by Philip et al.,) expressed at the CAR-T cell surface marked an important step forward. RQR8 has a dual role by acting simultaneously as a selection marker when CD34 is targeted with an anti-CD34 mAb (QBEnd/10) and as a suicide molecule when CD20 is targeted with rituximab. This epitope was engineered to match the Miltenyi CliniMACS system, and represented a breakthrough in terms of safety and the cost of generating clinical-grade CAR-T cells [47]. Although the preclinical studies are encouraging, this technology has not yet entered clinical development. In fact, rituximab’s mechanism of action means that it eliminates not only malignant B cells expressing CD20 but also normal B cells, which in turn interferes with the reconstitution of the B cell compartment after the injection of the CD20-elimination-marker-expressing CAR-T cells. These limitations might explain why this approach has not yet been trialed in the clinic. In contrast, the huEGFRt-cetuximab suicide switch (which also had good preclinical results [42,48]) has entered clinical trials. The mechanisms of action of the above-mentioned OFF-switch strategies are illustrated in Figure 5.

A study by Wang et al. showed that more than 50% of huEGFRt+ CAR-T cells were eliminated in vitro within an hour of cetuximab administration, and that these lymphocytes were depleted in NOD/*scid* mice after 4 to 6 days of daily intraperitoneal injection of 1 mg cetuximab [42]. Even though this approach to eradicating T cells may not be fast enough to reverse toxic effects in patients, huEGFRt+ CAR-T cells (approved under BB-IND-15829) are now being evaluated in many Phase I clinical trials targeting different types of cancer: relapsed/refractory CLL, NHL, ALL, multiple myeloma (MM), recurrent/refractory solid tumors, recurrent/refractory central nervous system (CNS) tumors, relapsed/refractory AML, persistent/recurrent blastic plasmacytoid dendritic cell neoplasm (BPDCN), and recurrent/refractory neuroblastoma. The corresponding clinical trials are summarized in Table 4. Given that no results have yet been published, it is too early to accurately predict whether this encouraging safety strategy will give good results in humans.

#### 2.1.3. SMASh CARs

**Figure 6:**
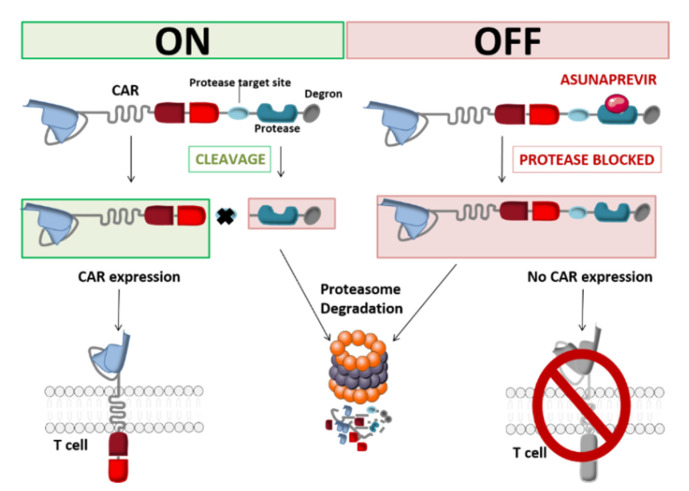
Schematic representation of the SWIFF-CAR principle (Adapted from Juillerat et al., 2019).

Another approach to protein elimination is small-molecule-assisted shutoff (SMASh). This strategy (developed by Chung et al.) provides reversible control of CAR production by incorporating a self-cleaving degradation moiety regulated by a protease/protease inhibitor pair. The CAR is bound to a drug-sensitive viral protease and a degron (a protein destabilization element) at the C-terminal end of the CAR. This construct is also known as a switch-off CAR (SWIFF-CAR) [49]. Juillerat et al. used the HCV NS3 protease and its inhibitor asunaprevir (ASN) to integrate this OFF-switch into CAR-T cells. In vitro and in the absence of ASN, the degron is cut off from the CAR and thus exposes the antigen-targeting scFv at the CAR-T cell surface. When ASN is added to the medium, HCV NS3-catalyzed cleavage of the degron is inhibited. This allows the CAR to be degraded by the T-cell’s proteolytic pathways (Figure 6). Using the small molecule ASN, the integration of a protease-based shut-off system into CAR-T cells can inhibit the latter’s action within 48 h [50].

In contrast to the two above-mentioned approaches, the switch-off CAR system is particular in that it can shut down the genetically engineered lymphocytes’ cytolytic properties without depleting the cells. In order to translate this OFF-switch system into the clinic and to determine its feasibility and utility in humans, intricate in vivo preclinical experiments are essential for evaluating basic properties like the relationship between the sparing of normal tissue and tumor control, switch-off triggering, engraftment, and proliferation [50]. Even though the clinical management of toxicity using suicide and elimination gene systems requires a direct response, non-lethal switch-off systems with slower off-kinetics might be a good substitute pending the results of more extensive studies. Their reversible, continuous control and cost-effectiveness are attractive features [51,52].

#### 2.1.4. The TKI-Based OFF-Switch

**Figure 7:**
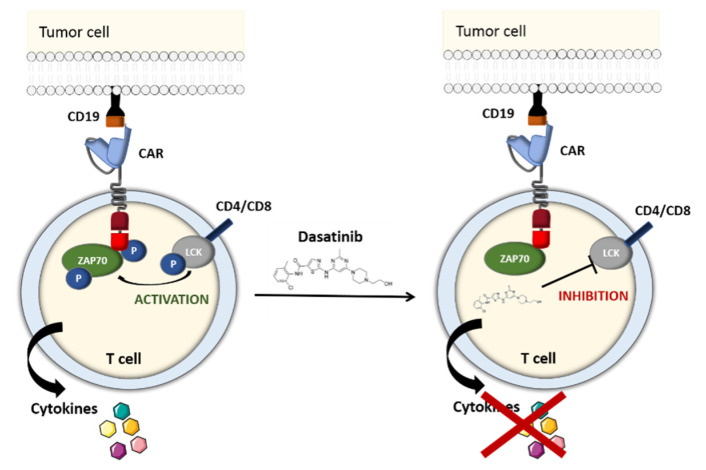
Schematic representation of the tyrosine kinase inhibitor (TKI)-based OFF-switch. The TKI dasatinib inhibits the phosphorylation of CD3ζ and ZAP70, which have a key role in the T-cell receptor (TCR) signaling pathway (Adapted from Wu et al., 2019).

Dasatinib is a selective BCR/ABL family tyrosine kinase inhibitor (TKI) that suppresses the activity of the uncontrolled tyrosine kinase ABL [53,54]. It also blocks the LCK kinase, which is primarily expressed in T cells. Hence, after having screened a panel of TKIs, Mestermann et al. suggested using dasatinib as a pharmacological OFF-switch for CAR-T cells. In Mestermann et al.’s subsequent co-culture study, CD4+ and CD8+ CD19 CAR-T cells were turned off by dasatinib in a dose-dependent manner. A Western blot analysis confirmed that the dasatinib prevented the phosphorylation of CD3ζ and zeta-chain-associated protein kinase 70 (ZAP70), which have key roles in the TCR signaling pathway and in the induction of NFAT—an important transcription factor in activated CAR-T cells (Figure 7). After a 2-h incubation, removal of the drug, and a 7-h latency period, the treated CAR-T cells recover their antitumor activity at much the same level as untreated CAR-T cells [55,56].

With the aim of examining dasatinib’s effectiveness and fast response in preventing life-threatening CRS, the groups led by Hudecek and Sadelain set up a murine preclinical model [55,57]. SCID/beige mice injected with Raji lymphoma cells were treated with CD19 CAR-T cells, which led to acute (and in some cases fatal) CRS during the first 48 h. Dasatinib administration resulted in a significant decrease (relative to control mice) in levels of interleukin-2 (IL-2), interferon gamma (IFNγ), tumor necrosis factor alfa (TNF-α) and granulocyte-macrophage colony-stimulating factor (GM-CSF) 3 h after CAR-T cell infusion. Forty-eight hours later, 70% of the TKI-treated mice and 25% of the control mice were still alive. The efficacy of reversible dasatinib-mediated control over CAR-T cell function was independent of the CAR’s target antigen (CD19, ROR1 or SLAMF7) and the costimulatory domain (CD28 or 4-1BB). All these encouraging results showed that in contrast to suicide and elimination gene systems, dasatinib is a potentially reversible pharmacological OFF/ON safety switch that can prevent fatal CRS without eradicating therapeutic CAR-T cells [55,58]. Further in vivo studies of this system are needed to confirm the functional control of CAR-T cells in a clinical setting.

### 2.2. CAR-T Cells with an ON-Switch

Another way to overcome potential toxicity is to develop therapeutic lymphocytes expressing an “ON-switch”. Instead of eliminating overreacting CAR-T cells that cause life-threatening conditions, the use of an activating “switch-on” molecule enables the precise remote control of these cells in terms of timing, site of action, and dosage. Wu et al. developed an inducible system in which the antigen-binding and intracellular signaling components cannot connect without the presence of an injectable small heterodimerizing molecule. This agent enables the in vivo activation of ON-switch CAR-T cells and controls the timing and the location of transgene expression—thus improving their safety profile. If serious adverse reaction occurs, the small molecule can be immediately discontinued and the expression of the specific CAR on T lymphocytes will return to baseline levels within a few hours or days, without the need to kill the cells [59].

#### 2.2.1. The Tetracycline-On System

Many preclinical studies have provided evidence of the potential advantages of the tetracycline-on (Tet-On) system [60]. This inducible gene expression system for mammalian cells is based on a reverse Tet transactivator (rtTA) fusion protein, which is responsible for the activation of the CAR gene promoter in the presence of doxycycline (Dox). The rtTA is composed of a doxycycline-binding Tet-repressor mutant protein and a C-terminal activator domain from the herpes simplex virus VP16 protein [61,62]. The first Tet-On systems needed two separate vectors: the first one held the rtTA gene and the second contained an inducible promoter for the desired gene. However, an all-in-one, third-generation Tet-inducible vector was recently proposed as an easier-to-use alternative for gene therapy [63].

For instance, Zhang et al. developed CD147 CAR-T cells with a tetracycline-controlled transcriptional activation system for the treatment of hepatocellular carcinoma. The researchers showed that CAR expression and function can be monitored with the Tet-On inducible gene system by Dox administration both in vitro and in vivo. The Tet-CD147 CAR’s expression level was highest 24 h after Dox administration and returned to basal values within 48 h of Dox removal from the culture medium. Nude mice bearing subcutaneous Huh-7 cells xenografts were then used to further evaluate the anti-tumor effect of Tet-CD147 CAR-T cells. The in vivo data showed that tumor volume and weight were lower in mice injected with (Dox+) Tet-CD147 CAR-T cells than in mice injected with (Dox−) Tet-CD147 CAR-T cells or peripheral blood mononuclear cells [64]. The Tet-On system has been tested in other inducible CAR-T cells, proving that this approach to controlling CAR -mediated activity has a better safety profile than conventional CAR-T cells but still has a powerful anti-tumor effect on B-cell malignancies (Tet-CD19 CAR-T cells) [65], and Tet-CD38 CAR-T cells for the treatment of MM [66]. Even though the aforementioned system is a promising toxicity-management strategy, further research is required to limit the adverse effects associated with the inducer; in fact, excessive exposure to Dox might accentuate the risk of antibiotic resistance in patients [67]. Clearly, the ideal solution would involve an alternative, non-antibiotic inducer molecule.

#### 2.2.2. Switchable Adaptor CARs

**Figure 8:**
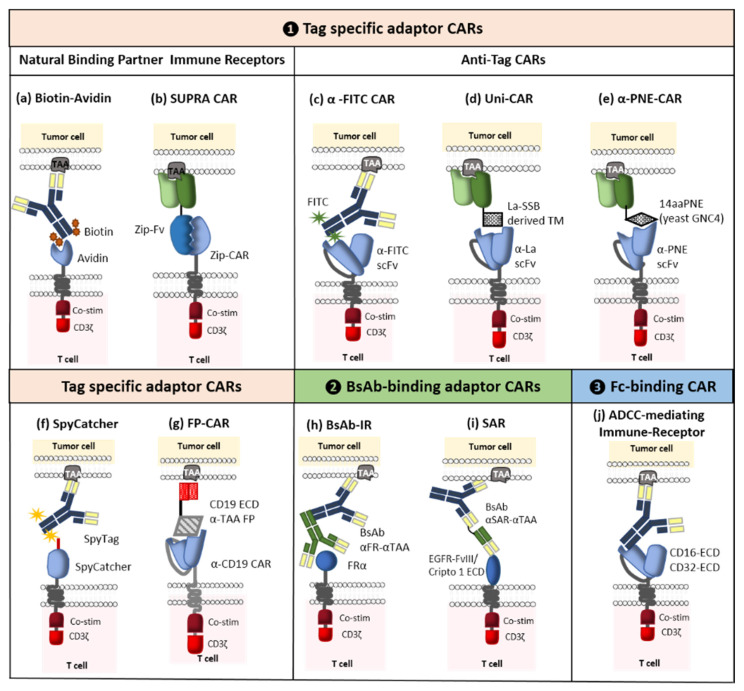
Schematic representation of switchable adaptors. (**1**) Tag-specific adaptors CARs: (**a**) biotin-binding immune receptors (BBIRs), (**b**) split, universal, and programmable (SUPRA) CARs, (**c**) α-FITC CARs, (**d**) UniCARs, (**e**) α-peptide-neoepitope (α-PNE) CARs, (**f**) SpyCatcher, (**g**) fusion protein (FP) CARs, (**2**) bispecific antibody-binding (BsAb) adaptor CARs: (**h**) BsAb immune receptors (BsAb-IRs), **(i**) synthetic agonistic receptors (SARs), (**3**) Fc-binding CARs: (**j**) ADCC-mediating immune receptor (Adapted from Arndt et al., 2020).

The concept of adaptor CARs was introduced in 2006 by Clemenceau et al. These molecular safety switches were designed to improve the flexibility, tumor specificity, and controllability of conventional CAR-T cells [68]. To this end, the tumor-targeting and signaling parts of the conventional CARs were separated. The resulting binary system comprised an adaptor CAR and various tumor-specific soluble adaptor molecules. Even though the CAR’s extracellular domain is connected to a binding partner that is inserted into the adaptor molecule (rather than a tumor-associated antigen), the adaptor CAR’s main structure is still analogous to a conventional CAR. In turn, the dual-function adaptor molecule connects the tumor and the CAR-T cell and thus enhances tumor specificity. Hence, the antitumor response is much the same as that induced by traditional CAR-T cells. This novel adaptability provides new options for solving the main problems posed by conventional CAR-T cell therapy, such as antigen-negative relapse. Indeed, a single adaptor CAR can activate engineered T cells against any type of target antigens. This strategy enables the production of universal CAR-T cells directed against a variety of TAAs, which eliminates the need for costly and arduous work developing new CARs and genetically modified T cells. Furthermore, the power and number of active CAR-T cells can be managed by controlling the concentration of the administered adaptor molecules. CAR-T cells which are not triggered by an adaptor molecule are silenced (i.e., they lack the ability to identify and kill the target cell). In due course, inactive cells will be eliminated by the body. Various researchers have designed ten or so diverse adaptor CAR platforms that can be further divided into three main categories: Tag-specific adaptor CARs, bispecific antibody (bsAb)-binding adaptor CARs, and constant fragment (Fc)-binding adaptor CARs (Figure 8). The first of these classes is the most developed and so we shall focus here on the corresponding clinical trials. For more information on the different categories of CAR, adaptor molecules, designs, side effects, and perspectives, see [69].

As mentioned above, Clémenceau et al. were the first to develop an adaptor CAR composed of the ECD of CD16 and the FcγRI receptor’s intracellular-signaling domain. It was well known that CD16 interacts with the Fc portion of immunoglobulin G (IgGs) [68]. These results encouraged other researchers to use this platform and develop first-generation CD16 CARs [70]. However, the first-generation CAR-T cells containing only the CD3ζ intracellular-signaling domain failed to trigger an efficient antitumor effect [71]; hence, the integration of costimulatory molecules like 4-1BB gave rise to second-generation CD16 CARs [72,73]. Activation of these CAR-T cells, requires the administration of tumor-specific mAbs (such as rituximab, cetuximab or trastuzumab) whose antigen binding fragment (Fab) can bind to the TAA and whose Fc part can bind to the CD16 CAR [68,72,73]. Moreover, Caratelli et al. suggested the use of the ECD from CD32A; CD16 binds to IgG2 with low affinity, whereas CD32A can bind to IgG1 and IgG2 [74]. One of the strengths of Fc-binding adaptor CAR-T cells is the broad range of mAbs able to mediate ADCC, which facilitates target switching during treatment. The mechanism of action of this ON-switch strategy is illustrated in Figure 8.

This new approach has been extensively tested in the clinic. The first adaptor CARs to enter clinical trials were ACTR087 (CD16-BB/ζ) and ACTR707 (CD16-28). Various mAbs were used to bridge the extracellular portion of the CD16 CARs to the antigen expressed on cancer cells: rituximab, trastuzumab, and the humanized non-fucosylated IgG1 mAb targeting B-cell maturation antigen (SEA-BCMA) for the treatment of CD20-positive B cell lymphoma, HER2-positive solid tumors, and BCMA-positive MM, respectively (Table 5).

A preliminary report on the Phase I ACTR087/rituximab clinical trial (NCT03189836) showed two complete responses and one partial response out of six evaluable patients with rituximab-resistant NHL treated with the low dose of 0.5 × 10^6^ ACTR T cells/kg, together with the anti-CD20 mAb rituximab. No adverse events were reported at this concentration. However, serious adverse events were noted for patients receiving a higher dose (1.5 × 10^6^/kg) of ACTR T cells. Two of nine evaluable patients died from severe CRS or neurotoxicity, which was (in some cases) considered to be related to ACTR087. As a consequence, the FDA placed a clinical hold on the trial [75]. After the clinical trial had resumed, it was again placed on hold because of grade 3 neurotoxicity, cytomegalovirus (CMV) infection, and grade 4 respiratory distress [76]. Furthermore, a dose-escalation Phase I clinical trial (NCT03680560) of the efficacy of ACTR707 CAR-T cells/kg with trastuzumab has been conducted and completed in patients with HER2-positive advanced cancers. In contrast to other ACTR707-based clinical studies, no adverse events or toxicities were reported [77]. The reasons behind these observed differences require further investigation.

Another category of switchable CAR is the tag-specific adaptor CAR. It is composed of an ECD that can identify and target an attached tag for tumor-specific adaptor molecules. This tag can be enzymatic, chemical, or genetic. Many subcategories have already been developed, including biotin-binding immune receptors [78], α-fluorescein isothiocyanate (α-FITC) CARs [79], UniCARs [80], α-peptide-neoepitope (PNE) CARs [81,82,83], split, universal, and programmable CARs [84], and a SpyCatcher CAR [85]. As with Fc-binding adaptor CARs, promising preclinical results have led to two Phase I clinical trial approvals. The first (ongoing) trial (NCT04230265) examines the effect of UniCAR-28/ζ (UniCAR02) T cells) administrated with CD123 transmembrane domains (TMs) in patients with CD123-positive hematologic and lymphatic cancers. The second trial has not started yet but will investigate the activity of a combination of α-PNE CAR-T cells (CLBR001) and a CD19 Fab switch (SWI019) in patients with relapsed/refractory B cell cancers [86]. Furthermore, α-FITC CARs with EC17 (folate-FITC) are advancing towards clinical development in an indication of osteosarcoma [69].

The third and final category corresponds to bsAb-binding adaptor CARs, which are able to simultaneously target two different antigens. This dual-targeting CAR-T cell strategy enables the use of a single type of genetically modified T cells with several bispecific adaptors designed to kill a wide variety of antigenically different solid tumors [87,88,89]. From 2014 onwards, scientific discoveries enabled the elaboration of three subcategories: bsAb-binding immune receptors (BsAb-IRs) [89], synthetic agonistic receptors (SARs) [90], and “integrated modules optimize adoptive cell therapy” strategies [91] (Figure 8). The popularity of this strategy prompted good manufacturing practice (GMP) production of the bifunctional adaptor molecule CD19ECD-αCD20, which will be used to treat relapsed CD19-negative cancer patients having previously received CD19 CAR-T cells; the goal is to reactivate these persistent CAR-T cells [92].

### 2.3. Strategies for Eliminating On-Target Off-Tumor Toxicity

**Figure 9:**
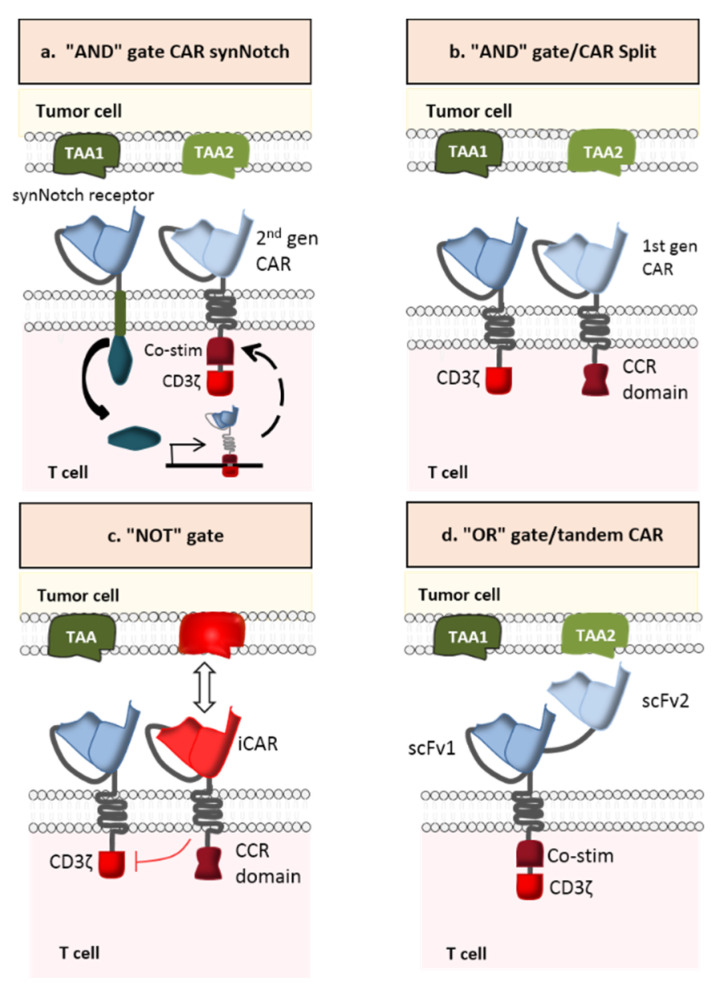
Combinatorial antigen targeting for solid tumors. (**a**) AND-gate CAR SynNotch (**b**) AND-gate/CAR Split, (**c**) NOT-gate CAR, and (**d**) OR-gate/tandem CAR (Adapted from Schmidts et al., 2018).

A novel strategy for preventing antigen escape (considered to be the major cause of resistance to cancer immunotherapy) is the multi-antigen-targeted CAR-T cell [93]. Various types have been developed: dual CAR-T cells, tandem CAR-T cells, trivalent CAR-T cells, and pooled CAR-T cells. The first type consists of combining two CARs that recognized different TAAs in a single CAR-T cell. The second type targets two TAAs with two distinct antigen-binding (scFv) domains linked in tandem to a single CAR. The third type contains three different CARs (recognizing three specific TAAs) in a single CAR-T cell. The last type is a combination of two CAR-T cell lines, each of which targets a specific TAA. Based on the signal transmission pattern, multi-antigen-targeted CAR-T cells have diverse logic-gate operations such as “AND”, “OR”, and “NOT” (Figure 9) [94].

Before we examine each strategy in detail, it is important to note that epitope selection is the most important determinant of therapeutic potential. As mentioned above, a valid TAA for CAR-T cells must meet rigorous criteria. However, neoantigens are considered to be the most attractive therapeutic targets in hematological and solid malignancies because (i) their expression is restricted to tumor cells that express the respective coding mutations and (ii) these antigens are recognized as non-self by the host immune system [95]. Most importantly, they meet the three main criteria for target selection in CAR-T cell therapy: coverage, stability, and specificity [93]. It is nevertheless difficult to find an ideal target for solid tumors due to inherent heterogeneity. This is why most of today’s targets cause significant off-target effects and limit the treatment’s intensity and effectiveness. Consequently, the treatment of solid tumors might require a combination of targets [93].

#### 2.3.1. AND-Gate CAR-T Cells

In contrast to OR-gate CARs, AND-gate CARs (dual CAR-T cells or trivalent CAR-T cells) have two or more extracellular domains activated solely by dual or triple antigen binding (Figure 9a,b). This strategy has been successfully tested in preclinical models of breast and prostate cancer [96,97]. In Kloss et al.’s preclinical study, CAR-T cells targeting prostate stem cell antigen (PSCA) and prostate-specific membrane antigen (PSMA) were generated in order to test the antitumor activity and safety profile of this combinatorial strategy. In fact, PSCA and PSMA are promising TAAs for prostate cancer [98] but neither is restricted to prostate tissue: PSCA is expressed in prostate tumors but also in the bladder, kidney, and other organs [99], while PSMA is expressed in metastatic prostate cancer but also in type II astrocytes, the kidney, and other organs [100]. CAR-T cells that display both antigen specificities were able to eradicate PSCA+/PSMA+ tumors in mice and thus achieve long-term survival of all treated animals. However, PSCA−/PSMA+ and PSCA+/PSMA− tumors were not destroyed, which demonstrates that using an AND logic gate can enhance the specificity and safety of CAR-T cell therapy [96].

It is important to note that the partial signaling derived from a single CAR can be strong enough to generate off-target toxicity [101]. This is why a more sophisticated approach (based on synthetic Notch (SynNotch) receptors) has been elaborated (Figure 9a). SynNotch receptors comprise the main regulatory domain of the cell-cell stimulating receptor Notch. However, the extracellular recognition domain and the intracellular transcriptional domain are both synthetic. Once the specific antigen binds to the synNotch receptor, an orthogonal transcription factor is released. It enters the nucleus and leads to the expression of another functional CAR, which targets a different TAA. Hence, local action is provided by the engineered lymphocyte and the customized gene expression program [102].

Further investigations of the immunogenicity of nonhuman transcription factors are required [103]. Preclinical studies have shown that synNotch CAR activation was only effective in dual-positive tumors; however, this was proof that spatial control had been achieved [104,105]. To prove the concept whereby synNotch receptors can control the expression of CARs, Roybal et al. engineered CAR-T cells with a CD19 synNotch receptor and α-mesothelin (MSLN) CAR gene. It is only when the T cell is exposed to CD19 that α-MSLN is expressed. The engineered lymphocytes are ready to be activated if the two TAAs are present. In light of the success of this approach, another experiment has been conducted using human primary CD4+ T cells engineered with a GFP synNotch receptor in which the CD19 CAR gene was inserted and whose expression is controlled by the corresponding response elements. These CAR-T cells were activated only upon contact with target cells expressing both GFP and CD19 on their surface [105]. Concerning AND-gate trivalent CAR-T cells, Sukumaran et al. demonstrated how genetically modified T cells expressing PSCA, TGFβ, and IL4 CARs can improve efficacy and safety in a pancreatic cancer model. These triple-specificity lymphocytes can reduce on-target/off-tumor effects by concentrating all their power on the cancer site. The preclinical studies of AND-gate CAR-T cells are summarized in Table 6.

#### 2.3.2. NOT-Gate CAR-T Cells

NOT-gate CAR-T cells (also referred to as inhibitory CAR (iCAR) T cells) contain both an activating receptor and an inhibitory receptor (Figure 9c). The interaction between the iCAR and its specific antigen induces an inhibitory signaling cascade and thus restrains T cell activity. This inhibitory receptor is engineered to target an antigen that is strongly expressed in healthy cells but almost absent in cancer cells—thus limiting the CAR-T cells’ access to tumor tissues. In a proof-of-concept study, Federov et al. developed an iCAR-T cell with two distinct receptors: a CD19 CAR linked to the CD28/CD3ζ signaling domain and a PSMA iCAR linked to an inhibitory intracellular domain derived either from programmed cell death-1 (PD-1) or cytotoxic T-lymphocyte-associated protein 4 (CTLA-4) antigens. The results proved the iCAR’s ability to exert negative control on T lymphocyte function when encountering cells co-expressing PSMA and CD19. In vitro PD-1-based iCAR-T cells were able to kill CD19+/PSMA- cells, since the absence of PSMA constitutes a “non-inhibitory” signal. In the presence of CD19+/PSMA+ cells, however, the cytotoxicity of iCAR-T cells was significantly reduced (by up to 95%). Cytokine secretion was also reduced (by up to 88%). In an in vivo mouse model, the data showed that PD-1-based iCAR-T cells (i) selectively inhibited the eradication of an “off-target” infused CD19+ B cell leukemia cell line (NALM/6) that expressed PSMA but (ii) efficiently killed “on-target” infused NALM/6 cells that did not express PSMA. Therefore, these NOT-gate CAR-T cells can distinguish between target and non-target cells in a heterogeneous population, both in vitro and in vivo [107]. Further research is needed to identify the most relevant combination of activating and inhibitory CARs and thus the ideal target antigens. In fact, identifying the right target for activating the iCAR is the major bottleneck in further development of iCAR-T cells.

#### 2.3.3. OR-Gate CAR-T Cells

Even though OR-gate CAR-T cells were originally engineered to avoid tumor resistance (rather than toxicity), we decided to develop this approach in the most promising next-generation CAR-T cells. OR-gate CAR-T cells can be activated in the presence of one of the targeted TAAs. It is now possible to develop a pool of specific dual, trivalent and tandem CAR-T cells by using the OR logic gate (Figure 9c). Pooled CAR-T cells targeting HER2/IL-13Rα2 for glioblastoma (GBM) [108], CD19/CD123 for B-ALL [109], and EGFR/CD133 [110] have been tested in preclinical studies and showed some benefits as well as some disadvantages. Although these CAR-T cells showed greater levels of cytolysis and cytokine secretion than the individual CAR-T cell lines, they were less efficacious than dual and tandem CAR-T cells. Furthermore, when monospecific CD19 and CD20 CAR-T cells were co-incubated with wild-type Raji cells, CD20 CAR-T cells proliferated significantly less than CD19 CAR-T cells—indicating that the combination of two CAR-T cell lines can lead to growth competition. The results also showed that this combination therapy exerts important immune pressure on the cancer cells, which may cause simultaneous tumor antigen escape [111]. Many clinical trials of this new approach have been initiated. In a Phase I clinical study (NCT02465983), patients with pancreatic cancer received a combination of mesothelin CAR-T cells and CD19 CAR-T cells. According to the investigators, this would increase the persistence of mesothelin-specific CAR-T cells in the body via the elimination of B cells by CD19 CAR-T cells and thus the elimination of antibodies against the CARs. Two other Phase I clinical trials used pooled CAR-T cell products consisting of CD19 and CD20 CAR-T cells for diffuse large B cell lymphoma (DLBCL) (NCT02737085), and CD19 and CD22 CAR-T cells for B-cell hematologic malignancies (NCT02903810), in order to effectively control recurrence due to a CD19 escape mutation. The known disadvantages of pooled CAR-T cells highlight the advantages of bispecific CAR-T cells. When, in the aforementioned trial, dual CAR-T cells targeting HER2/IL-13Rα2 encountered one of the two antigens, the intensity of signal transduction (ZAP-70 phosphorylation) was equal to that of the standard CAR-T cell. However, the intensity was greater when the dual CAR-T cells encounter both antigens simultaneously [108].

Many preclinical studies of tandem CAR-T cells using the OR logic gate have highlighted (i) the latter’s efficacy when at least one of the two antigens is recognized, and (ii) synergistic antitumor activity when both antigens are encountered [111,112,113,114]. It is also noteworthy that CD19/CD22 tandem CAR-T cells induced minimal residual disease-negative remission in a 22-year old patient with relapsed and refractory B-ALL after haploidentical HSCT. This clinical case highlights the tandem CAR-T cells’ potential for inducing long-term remission in B-ALL patients [115]. Like dual CAR-T cells, trivalent CAR-T cells have also shown potential antitumor activity. In a preclinical study, trivalent CAR-T cells (targeting IL13Rα2, EphA2, and HER2) were tested in order to overcome the antigen pair diversity seen in GBM patients and thus the insufficient antigen coverage of dual CAR-T cells. This experiment demonstrated that targeting three antigens with a single CAR-T cell can surmount interpatient antigen variability better than a bivalent combination, and can kill almost 100% of GBM cancer cells [116]. There are several explanations for this enhanced anticancer efficiency, including amplified signaling, a wider variety of targeted antigens, and stronger immunological synapse formation. Despite the above-mentioned advantages, OR-gate CAR-T cells are unfortunately associated with more on-target/off-tumor toxicity than AND- and NOT-gate CAR-T cells are. The preclinical studies of OR-gate CAR-T cells are summarized in Table 7.

## 3. Conclusions

Genetically modified T cells with synthetic immune receptors are exceptionally efficacious in the precision treatment of hematological cancers. However, this new era in cancer immunotherapy has only just started, and one can already envision significant advances in safety and efficacy in the coming years. In fact, one of the biggest obstacles to the development of CAR-T cells strategies is the potential toxicity due to overactivation of the immune system or on-target/off-tumor activation. Here, we reviewed the various innovative strategies developed to overcome these hurdles. Although many CAR-T cells have been efficacious in preclinical studies, few have entered clinical development. As mentioned above, some of these strategies (suicide genes and elimination markers) are designed to eliminate CAR-T cells quickly when toxicities occur, which permanently deactivates the long-term anticancer effect. Other approaches (SMASh CARs, the TKI-based OFF-switch, the Tet-ON system and switchable adaptor CARs) are able to reversibly turn off the CAR-T cells’ activity without eliminating these expensive and life-saving cell drugs. However, some of these deactivating approaches may take time to work and thus may lead to critical situations. Even though further research on the safety of CAR-T cells is required, one can legitimately hope that next-generation CAR-T cells will be safer as well as more efficacious. Gaining a better understanding of the obstacles posed by tumor heterogeneity and the tumor microenvironment should enable researchers to develop more precise strategies for immunosuppression and supportive care. Greater knowledge would also allow the engineered T lymphocytes to bypass the tumor’s immunosuppressive mechanisms. Other bottlenecks that must not be neglected are the practical and financial aspects of GMP CAR-T cell production—especially since the clinical trial results suggest that CAR-T cells and other genetically modified immune cells (such as CAR-NK cells) will be treatment options for broad range of cancers. Lastly, the rapid success of this strategy in oncology might prompt developments in other fields of medicine, such as the treatment of autoimmune as well as infectious disease.

## Figures and Tables

**Table 1:** Abbreviations.

Abbreviation	Referring to	Abbreviation	Referring to
ADCC	Antibody-dependent cellular cytotoxicity	HSV-TK	Herpes simplex virus thymidine kinase gene
ALL	Acute lymphoblastic leukemia	iCAR	Inhibitory CAR
AML	Acute myelogenous leukemia	iCasp9	Inducible caspase-9
ASN	Asunaprevir	IFNγ	Interferon gamma
BBIR	Biotin-binding immune receptor	IgG	Immunoglobulin G
BMT	Bone marrow transplantation	IL-2	Interleukin-2
BPDCN	Blastic plasmacytoid dendritic cell neoplasm	ITAM	Immunoreceptor tyrosine-based motif
BsAb	Bispecific antibody	mAb	Monoclonal antibody
BsAb-IR	BsAb immune receptor	MSLN	Mesothelin
CAR-T cell	Chimeric antigen receptor T cell	MHC	Major histocompatibility complex
CD	Cluster of differentiation	NHL	Non-Hodgkin’s lymphoma
CID	Chemical induction of dimerization	NK	Natural killer
CLL	Chronic lymphoblastic leukemia	PD-1	Programmed cell death-1
CNS	Central nervous system	PNE	Peptide neoepitope
CRS	Cytokine release syndrome	PSCA	Prostate stem cell antigen
CTLA-4	Cytotoxic T-lymphocyte-associated protein 4	PSMA	Prostate-specific membrane antigen
		rtTA	Reverse Tet transactivator
DIPG	Diffuse intrinsic pontine glioma	SAR	Synthetic agonistic receptor
DLBCL	Diffuse large B cell lymphoma	SWIFF-CAR	Switch-off CAR
DMG	Diffuse midline glioma	SMASh	Small-molecule-assisted shutoff
Dox	Doxycycline	SUPRA	Split, universal, and programmable
ECD	Extracellular domain	SynNotch	Synthetic Notch
EGFR	Epidermal growth factor receptor	TAA	Tumor-associated antigen
Fab	Fragment antigen binding	TCR	T cell receptor
FDA	Food and Drug Administration	Tet	Tetracycline
FITC	Fluorescein isothiocyanate	TKI	Tyrosine kinase inhibitor
GBM	Glioblastoma	TM	Transmembrane
GCV	Ganciclovir	TNF-α	Tumor necrosis factor alfa
GMP	Good manufacturing practice	TRUCK	T cells redirected for universal cytokine-mediated killing
GVHD	Graft-versus-host disease	TSA	Tumor-specific antigen
HER2	Human epidermal growth factor receptor-2	ZAP70	Zeta-chain-associated protein kinase 70

**Table 2:** Summary of preclinical studies of iCasp9 T cells co-expressing different CARs.

CAR-T Cells	Cancer Type	Time after Suicide Induction	Percentage of Eradicated CAR-T Cells	Reference
iCasp9-IL15-CD19CAR	B-cell malignancies	24 h	>95.0%	[35]
iCasp9-CD20CAR-Δ19	B-cell malignancies	24 h 72 h	90.0% 98.0%	[36]
iCasp9-CD20CAR-ΔNCFR	B-cell malignancies	48 h	>99.0%	[37]
iCasp9-CD33CAR-Δ19	AML	N/A	76.4%	[38]
iCasp9-IL1RAPCAR-Δ19	CML	N/A	87.0%	[39]

**Table 3:** Summary of clinical trials using iCasp9 CAR-T cells.

Target	Cancer Types	NCT Number
GD2	DIPG and DMG	NCT04196413
	Relapsed/refractory neuroblastoma	NCT01822652 NCT03721068 NCT03373097
	Refractory/metastatic GD2-positive sarcoma or neuroblastoma	NCT01953900 NCT02107963
	Solid cancers	NCT02992210
CD19	Relapsed/refractory ALL	NCT03016377 NCT03594162
	Relapsed/refractory B-cell lymphoma	NCT03696784 NCT03579927
CD19 & CD22	Relapsed/refractory B-cell lymphoma	NCT03098355
CD30	Relapsed/refractory CD30-positive lymphomas	NCT02274584
Mesothelin	Solid cancers	NCT02414269
	Advanced breast cancer	NCT02792114

**Table 4:** Summary of clinical trials of huEGFRt+ CAR-T cells.

Target	Cancer Types	NCT Number
CD19	CLL, NHL and ALL	NCT01865617 NCT03103971 NCT01815749 NCT03085173 NCT02146924 NCT02051257 NCT03579888 NCT02028455 NCT02746952
EGFR	Recurrent/refractory solid tumors	NCT03618381
	Recurrent or refractory pediatric CNS tumors	NCT03638167
HER2	HER2-positive recurrent/refractory pediatric CNS tumors	NCT03500991
BCMA	MM	NCT03070327
CD22	CD22+ leukemia and lymphoma	NCT03244306
CD123	CD123+ relapsed/refractory AML or persistent/recurrent BPDCN	NCT03114670 NCT02159495
CD171	Recurrent/refractory neuroblastoma or ganglioneuroblastoma	NCT02311621
PD-1	Recurrent glioblastoma multiforme	NCT02937844

**Table 5:** Summary of clinical trials using Fc-binding adaptor CARs.

CAR Adaptor	Adaptor Molecule	Cancer Type	NCT Number
CD16-BB/ζ (ACTR087)	Rituximab	Refractory or relapsed CD20-positive B cell lymphoma	NCT02776813
CD16-28 (ACTR707)			NCT03189836
ACTR087 ACTR707	Trastuzumab	HER2-positive advanced solid tumor cancers	NCT03680560
ACTR087 ACTR707	Rituximab Trastuzumab SEA-BCMA	B cell lymphoma MM HER2-positive solid tumor cancers	NCT02840110
ACTR087	SEA-BCMA	Relapsed or refractory MM	NCT03266692

**Table 6:** Preclinical studies of AND-gate CAR-T cells.

CAR-T Cell Type	Target	Cancer Types	Reference
Dual CAR-T cell	PSMA/PSCA	Prostate cancer	[96]
	FRa/MSLN	Ovarian cancer	[101]
	CD19/GFP	Experimental cancer	[105]
	CD19/MSLN	Experimental cancer	[105]
Trivalent CAR-T cell	PSCA/TGFβ/IL4	Pancreatic cancer	[106]

**Table 7:** Summary of preclinical studies of OR-gate CAR-T cells.

CAR-T Cell Type	Target	Cancer Type	Reference
Pooled CAR-T cell	HER2/IL-13Rα2	GMB	[108]
	CD19/CD123	B-ALL	[109]
	CD19/CD20	B-ALL	[111]
	EGFR/CD133	Cholangiocarcinoma	[110]
Dual CAR-T cell	CD19/CD123	B-ALL	[109]
	HER2/IL-13Rα2	GMB	[108]
Tandem CAR-T cell	CD19/CD20	B cell malignancies	[111]
	HER2/IL-13Rα2	GMB	[108]
	CD20/HER2	Experimental Cancer	[114]
	CD19/HER2	Experimental Cancer	[112]
Trivalent CAR-T cell	HER2/IL13Rα2/EphA2	GMB	[116]

## References

[1] McCarthy E.F. (2006). The Toxins of William B. Coley and the Treatment of Bone and Soft-Tissue Sarcomas. Iowa Orthop. J..

[2] Köhler G., Milstein C. (1975). Continuous cultures of fused cells secreting antibody of predefined specificity. Nature.

[3] Mach J.-P. (2012). Introduction to monoclonal antibodies. Cancer Immun..

[4] Eshhar Z., Waks T., Gross G., Schindler D.G. (1993). Specific activation and targeting of cytotoxic lymphocytes through chimeric single chains consisting of antibody-binding domains and the gamma or zeta subunits of the immunoglobulin and T-cell receptors. Proc. Natl. Acad. Sci. USA.

[5] Golay J., Andrea A.E. (2020). Combined Anti-Cancer Strategies Based on Anti-Checkpoint Inhibitor Antibodies. Antibodies.

[6] Irving B.A., Weiss A. (1991). The cytoplasmic domain of the T cell receptor ζ chain is sufficient to couple to receptor-associated signal transduction pathways. Cell.

[7] Lee Y.-H., Kim C.H. (2019). Evolution of chimeric antigen receptor (CAR) T cell therapy: Current status and future perspectives. Arch. Pharm. Res..

[8] Letourneur F., Klausner R.D. (1992). Activation of T cells by a tyrosine kinase activation domain in the cytoplasmic tail of CD3 epsilon. Science.

[9] Romeo C., Amiot M., Seed B. (1992). Sequence requirements for induction of cytolysis by the T cell antigen/Fc receptor zeta chain. Cell.

[10] Klampatsa A., Haas A.R., Moon E.K., Albelda S.M. (2017). Chimeric Antigen Receptor (CAR) T Cell Therapy for Malignant Pleural Mesothelioma (MPM). Cancers.

[11] Marincola F.M., Jaffee E.M., Hicklin D.J., Ferrone S. (2000). Escape of human solid tumors from T-cell recognition: Molecular mechanisms and functional significance. Adv. Immunol..

[12] Garrido F., Algarra I. (2001). MHC antigens and tumor escape from immune surveillance. Adv. Cancer Res..

[13] Cabrera T., López-Nevot M.A., Gaforio J.J., Ruiz-Cabello F., Garrido F. (2003). Analysis of HLA expression in human tumor tissues. Cancer Immunol. Immunother..

[14] Barrett D.M., Grupp S.A., June C.H. (2015). Chimeric Antigen Receptor– and TCR-Modified T Cells Enter Main Street and Wall Street. J. Immunol..

[15] Fesnak A.D., Levine B.L., June C.H. (2016). Engineered T Cells: The Promise and Challenges of Cancer Immunotherapy. Nat. Rev. Cancer.

[16] Brudno J.N., Kochenderfer J.N. (2016). Toxicities of chimeric antigen receptor T cells: Recognition and management. Blood.

[17] Kulemzin S.V., Kuznetsova V.V., Mamonkin M., Taranin A.V., Gorchakov A.A. (2017). CAR T-cell therapy: Balance of efficacy and safety. Mol. Biol. (Mosk.).

[18] Brentjens R.J., Santos E., Nikhamin Y., Yeh R., Matsushita M., La Perle K., Quintás-Cardama A., Larson S.M., Sadelain M. (2007). Genetically targeted T cells eradicate systemic acute lymphoblastic leukemia xenografts. Clin. Cancer Res..

[19] Finney H.M., Akbar A.N., Lawson A.D.G. (2004). Activation of resting human primary T cells with chimeric receptors: Costimulation from CD28, inducible costimulator, CD134, and CD137 in series with signals from the TCR zeta chain. J. Immunol..

[20] Imai C., Mihara K., Andreansky M., Nicholson I.C., Pui C.-H., Geiger T.L., Campana D. (2004). Chimeric receptors with 4-1BB signaling capacity provoke potent cytotoxicity against acute lymphoblastic leukemia. Leukemia.

[21] Song D.-G., Ye Q., Poussin M., Harms G.M., Figini M., Powell D.J. (2012). CD27 costimulation augments the survival and antitumor activity of redirected human T cells in vivo. Blood.

[22] Smith A.J., Oertle J., Warren D., Prato D. (2016). Chimeric antigen receptor (CAR) T cell therapy for malignant cancers: Summary and perspective. J. Cell. Immunother..

[23] Li H., Zhao Y. (2017). Increasing the safety and efficacy of chimeric antigen receptor T cell therapy. Protein Cell.

[24] Maude S.L., Teachey D.T., Porter D.L., Grupp S.A. (2015). CD19-targeted chimeric antigen receptor T-cell therapy for acute lymphoblastic leukemia. Blood.

[25] Morgan R.A., Yang J.C., Kitano M., Dudley M.E., Laurencot C.M., Rosenberg S.A. (2010). Case report of a serious adverse event following the administration of T cells transduced with a chimeric antigen receptor recognizing ERBB2. Mol. Ther..

[26] Fillat C., Carrió M., Cascante A., Sangro B. (2003). Suicide gene therapy mediated by the Herpes Simplex virus thymidine kinase gene/Ganciclovir system: Fifteen years of application. Curr. Gene Ther..

[27] Ciceri F., Bonini C., Marktel S., Zappone E., Servida P., Bernardi M., Pescarollo A., Bondanza A., Peccatori J., Rossini S. (2007). Antitumor effects of HSV-TK-engineered donor lymphocytes after allogeneic stem-cell transplantation. Blood.

[28] Greco R., Oliveira G., Stanghellini M.T.L., Vago L., Bondanza A., Peccatori J., Cieri N., Marktel S., Mastaglio S., Bordignon C. (2015). Improving the safety of cell therapy with the TK-suicide gene. Front. Pharmacol..

[29] Beltinger C., Fulda S., Kammertoens T., Meyer E., Uckert W., Debatin K.M. (1999). Herpes simplex virus thymidine kinase/ganciclovir-induced apoptosis involves ligand-independent death receptor aggregation and activation of caspases. Proc. Natl. Acad. Sci. USA.

[30] Traversari C., Marktel S., Magnani Z., Mangia P., Russo V., Ciceri F., Bonini C., Bordignon C. (2007). The potential immunogenicity of the TK suicide gene does not prevent full clinical benefit associated with the use of TK-transduced donor lymphocytes in HSCT for hematologic malignancies. Blood.

[31] Tiberghien P., Ferrand C., Lioure B., Milpied N., Angonin R., Deconinck E., Certoux J.M., Robinet E., Saas P., Petracca B. (2001). Administration of herpes simplex-thymidine kinase-expressing donor T cells with a T-cell-depleted allogeneic marrow graft. Blood.

[32] Ciceri F., Bonini C., Stanghellini M.T.L., Bondanza A., Traversari C., Salomoni M., Turchetto L., Colombi S., Bernardi M., Peccatori J. (2009). Infusion of suicide-gene-engineered donor lymphocytes after family haploidentical haemopoietic stem-cell transplantation for leukaemia (the TK007 trial): A non-randomised phase I-II study. Lancet Oncol..

[33] Straathof K.C., Pulè M.A., Yotnda P., Dotti G., Vanin E.F., Brenner M.K., Heslop H.E., Spencer D.M., Rooney C.M. (2005). An inducible caspase 9 safety switch for T-cell therapy. Blood.

[34] Di Stasi A., Tey S.-K., Dotti G., Fujita Y., Kennedy-Nasser A., Martinez C., Straathof K., Liu E., Durett A.G., Grilley B. (2011). Inducible apoptosis as a safety switch for adoptive cell therapy. N. Engl. J. Med..

[35] Hoyos V., Savoldo B., Quintarelli C., Mahendravada A., Zhang M., Vera J., Heslop H.E., Rooney C.M., Brenner M.K., Dotti G. (2010). Engineering CD19-specific T lymphocytes with interleukin-15 and a suicide gene to enhance their anti-lymphoma/leukemia effects and safety. Leukemia.

[36] Budde L.E., Berger C., Lin Y., Wang J., Lin X., Frayo S.E., Brouns S.A., Spencer D.M., Till B.G., Jensen M.C. (2013). Combining a CD20 Chimeric Antigen Receptor and an Inducible Caspase 9 Suicide Switch to Improve the Efficacy and Safety of T Cell Adoptive Immunotherapy for Lymphoma. PLoS ONE.

[37] Diaconu I., Ballard B., Zhang M., Chen Y., West J., Dotti G., Savoldo B. (2017). Inducible Caspase-9 Selectively Modulates the Toxicities of CD19-Specific Chimeric Antigen Receptor-Modified T Cells. Mol. Ther..

[38] Minagawa K., Jamil M.O., Al-Obaidi M., Pereboeva L., Salzman D., Erba H.P., Lamb L.S., Bhatia R., Mineishi S., Di Stasi A. (2016). In Vitro Pre-Clinical Validation of Suicide Gene Modified Anti-CD33 Redirected Chimeric Antigen Receptor T-Cells for Acute Myeloid Leukemia. PLoS ONE.

[39] Warda W., Larosa F., Neto Da Rocha M., Trad R., Deconinck E., Fajloun Z., Faure C., Caillot D., Moldovan M., Valmary-Degano S. (2019). CML Hematopoietic Stem Cells Expressing IL1RAP Can Be Targeted by Chimeric Antigen Receptor-Engineered T Cells. Cancer Res..

[40] Griffioen M., van Egmond E.H.M., Kester M.G.D., Willemze R., Falkenburg J.H.F., Heemskerk M.H.M. (2009). Retroviral transfer of human CD20 as a suicide gene for adoptive T-cell therapy. Haematologica.

[41] Vogler I., Newrzela S., Hartmann S., Schneider N., von Laer D., Koehl U., Grez M. (2010). An Improved Bicistronic CD20/tCD34 Vector for Efficient Purification and In Vivo Depletion of Gene-Modified T Cells for Adoptive Immunotherapy. Mol. Ther..

[42] Wang X., Chang W.-C., Wong C.W., Colcher D., Sherman M., Ostberg J.R., Forman S.J., Riddell S.R., Jensen M.C. (2011). A transgene-encoded cell surface polypeptide for selection, in vivo tracking, and ablation of engineered cells. Blood.

[43] Paszkiewicz P.J., Fräßle S.P., Srivastava S., Sommermeyer D., Hudecek M., Drexler I., Sadelain M., Liu L., Jensen M.C., Riddell S.R. (2016). Targeted antibody-mediated depletion of murine CD19 CAR T cells permanently reverses B cell aplasia. J. Clin. Investig..

[44] Kieback E., Charo J., Sommermeyer D., Blankenstein T., Uckert W. (2008). A safeguard eliminates T cell receptor gene-modified autoreactive T cells after adoptive transfer. Proc. Natl. Acad. Sci. USA.

[45] Introna M., Barbui A.M., Bambacioni F., Casati C., Gaipa G., Borleri G., Bernasconi S., Barbui T., Golay J., Biondi A. (2000). Genetic modification of human T cells with CD20: A strategy to purify and lyse transduced cells with anti-CD20 antibodies. Hum. Gene Ther..

[46] Serafini M., Manganini M., Borleri G., Bonamino M., Imberti L., Biondi A., Golay J., Rambaldi A., Introna M. (2004). Characterization of CD20-transduced T lymphocytes as an alternative suicide gene therapy approach for the treatment of graft-versus-host disease. Hum. Gene Ther..

[47] Philip B., Kokalaki E., Mekkaoui L., Thomas S., Straathof K., Flutter B., Marin V., Marafioti T., Chakraverty R., Linch D. (2014). A highly compact epitope-based marker/suicide gene for easier and safer T-cell therapy. Blood.

[48] Koneru M., Purdon T.J., Spriggs D., Koneru S., Brentjens R.J. (2015). IL-12 secreting tumor-targeted chimeric antigen receptor T cells eradicate ovarian tumors in vivo. Oncoimmunology.

[49] Chung H.K., Jacobs C.L., Huo Y., Yang J., Krumm S.A., Plemper R.K., Tsien R.Y., Lin M.Z. (2015). Tunable and reversible drug control of protein production via a self-excising degron. Nat. Chem. Biol..

[50] Juillerat A., Tkach D., Busser B.W., Temburni S., Valton J., Duclert A., Poirot L., Depil S., Duchateau P. (2019). Modulation of chimeric antigen receptor surface expression by a small molecule switch. BMC Biotechnol..

[51] Valton J., Guyot V., Boldajipour B., Sommer C., Pertel T., Juillerat A., Duclert A., Sasu B.J., Duchateau P., Poirot L. (2018). A Versatile Safeguard for Chimeric Antigen Receptor T-Cell Immunotherapies. Sci. Rep..

[52] Casucci M., Bondanza A. (2011). Suicide Gene Therapy to Increase the Safety of Chimeric Antigen Receptor-Redirected T Lymphocytes. J. Cancer.

[53] Khoury H.J., Guilhot F., Hughes T.P., Kim D.-W., Cortes J.E. (2009). Dasatinib treatment for Philadelphia chromosome-positive leukemias: Practical considerations. Cancer.

[54] Conchon M., Freitas C.M.B.d.M., Rego M.A.d.C., Braga Junior J.W.R. (2011). Dasatinib-clinical trials and management of adverse events in imatinib resistant/intolerant chronic myeloid leukemia. Rev. Bras. Hematol. Hemoter.

[55] Mestermann K., Giavridis T., Weber J., Rydzek J., Frenz S., Nerreter T., Mades A., Sadelain M., Einsele H., Hudecek M. (2019). The tyrosine kinase inhibitor dasatinib acts as a pharmacologic on/off switch for CAR T cells. Sci. Transl. Med..

[56] Rydzek J., Nerreter T., Peng H., Jutz S., Leitner J., Steinberger P., Einsele H., Rader C., Hudecek M. (2019). Chimeric Antigen Receptor Library Screening Using a Novel NF-κB/NFAT Reporter Cell Platform. Mol. Ther..

[57] Giavridis T., van der Stegen S.J.C., Eyquem J., Hamieh M., Piersigilli A., Sadelain M. (2018). CAR T cell-induced cytokine release syndrome is mediated by macrophages and abated by IL-1 blockade. Nat. Med..

[58] Wu B.X., Song N.-J., Riesenberg B.P., Li Z. (2019). Development of molecular and pharmacological switches for chimeric antigen receptor T cells. Exp. Hematol. Oncol..

[59] Wu C.-Y., Roybal K.T., Puchner E.M., Onuffer J., Lim W.A. (2015). Remote control of therapeutic T cells through a small molecule-gated chimeric receptor. Science.

[60] Loew R., Heinz N., Hampf M., Bujard H., Gossen M. (2010). Improved Tet-responsive promoters with minimized background expression. BMC Biotechnol..

[61] Gossen M., Bujard H. (1992). Tight control of gene expression in mammalian cells by tetracycline-responsive promoters. Proc. Natl. Acad. Sci. USA.

[62] Urlinger S., Baron U., Thellmann M., Hasan M.T., Bujard H., Hillen W. (2000). Exploring the sequence space for tetracycline-dependent transcriptional activators: Novel mutations yield expanded range and sensitivity. Proc. Natl. Acad. Sci. USA.

[63] Heinz N., Schambach A., Galla M., Maetzig T., Baum C., Loew R., Schiedlmeier B. (2011). Retroviral and transposon-based tet-regulated all-in-one vectors with reduced background expression and improved dynamic range. Hum. Gene Ther..

[64] Zhang R.-Y., Wei D., Liu Z.-K., Yong Y.-L., Wei W., Zhang Z.-Y., Lv J.-J., Zhang Z., Chen Z.-N., Bian H. (2019). Doxycycline Inducible Chimeric Antigen Receptor T Cells Targeting CD147 for Hepatocellular Carcinoma Therapy. Front. Cell Dev. Biol..

[65] Gu X., He D., Li C., Wang H., Yang G. (2018). Development of Inducible CD19-CAR T Cells with a Tet-On System for Controlled Activity and Enhanced Clinical Safety. Int J. Mol. Sci.

[66] Drent E., Poels R., Mulders M.J., van de Donk N.W.C.J., Themeli M., Lokhorst H.M., Mutis T. (2018). Feasibility of controlling CD38-CAR T cell activity with a Tet-on inducible CAR design. PLoS ONE.

[67] González-Zorn B., Escudero J.A. (2012). Ecology of antimicrobial resistance: Humans, animals, food and environment. Int. Microbiol..

[68] Clémenceau B., Congy-Jolivet N., Gallot G., Vivien R., Gaschet J., Thibault G., Vié H. (2006). Antibody-dependent cellular cytotoxicity (ADCC) is mediated by genetically modified antigen-specific human T lymphocytes. Blood.

[69] Arndt C., Fasslrinner F., Loureiro L.R., Koristka S., Feldmann A., Bachmann M. (2020). Adaptor CAR Platforms—Next Generation of T Cell-Based Cancer Immunotherapy. Cancers.

[70] Tanaka H., Fujiwara H., Ochi F., Tanimoto K., Casey N., Okamoto S., Mineno J., Kuzushima K., Shiku H., Sugiyama T. (2016). Development of Engineered T Cells Expressing a Chimeric CD16-CD3ζ Receptor to Improve the Clinical Efficacy of Mogamulizumab Therapy Against Adult T-Cell Leukemia. Clin. Cancer Res..

[71] Bridgeman J.S., Hawkins R.E., Hombach A.A., Abken H., Gilham D.E. (2010). Building better chimeric antigen receptors for adoptive T cell therapy. Curr. Gene Ther..

[72] Kudo K., Imai C., Lorenzini P., Kamiya T., Kono K., Davidoff A.M., Chng W.J., Campana D. (2014). T Lymphocytes Expressing a CD16 Signaling Receptor Exert Antibody-Dependent Cancer Cell Killing. Cancer Res..

[73] Rataj F., Jacobi S.J., Stoiber S., Asang F., Ogonek J., Tokarew N., Cadilha B.L., van Puijenbroek E., Heise C., Duewell P. (2019). High-affinity CD16-polymorphism and Fc-engineered antibodies enable activity of CD16-chimeric antigen receptor-modified T cells for cancer therapy. Br. J. Cancer.

[74] Caratelli S., Arriga R., Sconocchia T., Ottaviani A., Lanzilli G., Pastore D., Cenciarelli C., Venditti A., Del Principe M.I., Lauro D. (2020). In vitro elimination of epidermal growth factor receptor-overexpressing cancer cells by CD32A-chimeric receptor T cells in combination with cetuximab or panitumumab. Int. J. Cancer.

[75] Minutolo N.G., Hollander E.E., Powell D.J.J. (2019). The Emergence of Universal Immune Receptor T Cell Therapy for Cancer. Front. Oncol..

[76] FDA Places Clinical Hold on Unum Therapeutics Phase 1 Trial Evaluating ACTR087 for Relapsed/Refractory CD20+ B Cell Non-Hodgkin Lymphoma. https://www.trialsitenews.com/fda-places-clinical-hold-on-unum-therapeutics-phase-1-trial-evaluating-actr087-for-relapsed-refractory-cd20-b-cell-non-hodgkin-lymphoma/.

[77] Unum Therapeutics Inc. Unum Therapeutics Provides Updates to Its Phase 1 Trial of ACTR707 for HER2+ Solid Tumor Cancers. http://www.globenewswire.com/news-release/2020/01/29/1976692/0/en/Unum-Therapeutics-Provides-Updates-to-its-Phase-1-Trial-of-ACTR707-for-HER2-Solid-Tumor-Cancers.html.

[78] Urbanska K., Lanitis E., Poussin M., Lynn R.C., Gavin B.P., Kelderman S., Yu J., Scholler N., Powell D.J. (2012). A Universal Strategy for Adoptive Immunotherapy of Cancer through Use of a Novel T-cell Antigen Receptor. Cancer Res..

[79] Tamada K., Geng D., Sakoda Y., Bansal N., Srivastava R., Li Z., Davila E. (2012). Redirecting gene-modified T cells toward various cancer types using tagged antibodies. Clin. Cancer Res..

[80] Cartellieri M., Loff S., von Bonin M., Bejestani E.P., Ehninger A., Feldmann A., Koristka S., Arndt C., Ehninger G., Bachmann M.P. (2015). Unicar: A Novel Modular Retargeting Platform Technology for CAR T Cells. Blood.

[81] Rodgers D.T., Mazagova M., Hampton E.N., Cao Y., Ramadoss N.S., Hardy I.R., Schulman A., Du J., Wang F., Singer O. (2016). Switch-mediated activation and retargeting of CAR-T cells for B-cell malignancies. Proc. Natl. Acad. Sci. USA.

[82] Raj D., Yang M.-H., Rodgers D., Hampton E.N., Begum J., Mustafa A., Lorizio D., Garces I., Propper D., Kench J.G. (2019). Switchable CAR-T cells mediate remission in metastatic pancreatic ductal adenocarcinoma. Gut.

[83] Viaud S., Ma J.S.Y., Hardy I.R., Hampton E.N., Benish B., Sherwood L., Nunez V., Ackerman C.J., Khialeeva E., Weglarz M. (2018). Switchable control over in vivo CAR T expansion, B cell depletion, and induction of memory. Proc. Natl. Acad. Sci. USA.

[84] Cho J.H., Collins J.J., Wong W.W. (2018). Universal Chimeric Antigen Receptors for Multiplexed and Logical Control of T Cell Responses. Cell.

[85] Minutolo N.G., Sharma P., Poussin M., Shaw L.C., Brown D.P., Hollander E.E., Smole A., Rodriguez-Garcia A., Hui J.Z., Zappala F. (2020). Quantitative Control of Gene-Engineered T-Cell Activity through the Covalent Attachment of Targeting Ligands to a Universal Immune Receptor. J. Am. Chem. Soc..

[86] AbbVie & Scripps-based Calibr Moves Novel ‘Switchable’ CAR-T Technology to Phase I Clinical Trial. https://www.trialsitenews.com/abbvie-scripps-based-calibr-moves-novel-switchable-car-t-technology-to-phase-i-clinical-trial/.

[87] Stamova S., Koristka S., Keil J., Arndt C., Feldmann A., Michalk I., Bartsch H., Bippes C.C., Schmitz M., Cartellieri M. (2012). Cancer Immunotherapy by Retargeting of Immune Effector Cells via Recombinant Bispecific Antibody Constructs. Antibodies.

[88] Lee Y.G., Marks I., Srinivasarao M., Kanduluru A.K., Mahalingam S.M., Liu X., Chu H., Low P.S. (2019). Use of a Single CAR T Cell and Several Bispecific Adapters Facilitates Eradication of Multiple Antigenically Different Solid Tumors. Cancer Res..

[89] Urbanska K., Lynn R.C., Stashwick C., Thakur A., Lum L.G., Powell D.J. (2014). Targeted cancer immunotherapy via combination of designer bispecific antibody and novel gene-engineered T cells. J. Transl. Med..

[90] Karches C.H., Benmebarek M.-R., Schmidbauer M.L., Kurzay M., Klaus R., Geiger M., Rataj F., Cadilha B.L., Lesch S., Heise C. (2019). Bispecific Antibodies Enable Synthetic Agonistic Receptor-Transduced T Cells for Tumor Immunotherapy. Clin. Cancer Res..

[91] Ambrose C., Su L., Wu L., Lobb R.R., Rennert P.D. (2017). Abstract 3768: CAR T cells specific for CD19 can be redirected to kill CD19 negative tumors. Cancer Res..

[92] Rennert P., Su L., Dufort F., Birt A., Sanford T., Wu L., Ambrose C., Lobb R. (2019). A Novel CD19-Anti-CD20 Bridging Protein Prevents and Reverses CD19-Negative Relapse from CAR19 T Cell Treatment In Vivo. Blood.

[93] Wei J., Han X., Bo J., Han W. (2019). Target selection for CAR-T therapy. J. Hematol. Oncol..

[94] Han X., Wang Y., Wei J., Han W. (2019). Multi-antigen-targeted chimeric antigen receptor T cells for cancer therapy. J. Hematol. Oncol..

[95] Schumacher T.N., Scheper W., Kvistborg P. (2019). Cancer Neoantigens. Annu. Rev. Immunol..

[96] Kloss C.C., Condomines M., Cartellieri M., Bachmann M., Sadelain M. (2013). Combinatorial antigen recognition with balanced signaling promotes selective tumor eradication by engineered T cells. Nat. Biotechnol..

[97] Wilkie S., van Schalkwyk M.C.I., Hobbs S., Davies D.M., van der Stegen S.J.C., Pereira A.C.P., Burbridge S.E., Box C., Eccles S.A., Maher J. (2012). Dual targeting of ErbB2 and MUC1 in breast cancer using chimeric antigen receptors engineered to provide complementary signaling. J. Clin. Immunol..

[98] Feldmann A., Arndt C., Bergmann R., Loff S., Cartellieri M., Bachmann D., Aliperta R., Hetzenecker M., Ludwig F., Albert S. (2017). Retargeting of T lymphocytes to PSCA- or PSMA positive prostate cancer cells using the novel modular chimeric antigen receptor platform technology “UniCAR”. Oncotarget.

[99] Lam J.S., Yamashiro J., Shintaku I.P., Vessella R.L., Jenkins R.B., Horvath S., Said J.W., Reiter R.E. (2005). Prostate stem cell antigen is overexpressed in prostate cancer metastases. Clin. Cancer Res..

[100] Silver D.A., Pellicer I., Fair W.R., Heston W.D., Cordon-Cardo C. (1997). Prostate-specific membrane antigen expression in normal and malignant human tissues. Clin. Cancer Res..

[101] Lanitis E., Poussin M., Klattenhoff A.W., Song D., Sandaltzopoulos R., June C.H., Powell D.J. (2013). Chimeric Antigen Receptor T Cells with Dissociated Signaling Domains Exhibit Focused Antitumor Activity with Reduced Potential for Toxicity In Vivo. Cancer Immunol. Res..

[102] Roybal K.T., Williams J.Z., Morsut L., Rupp L.J., Kolinko I., Choe J.H., Walker W.J., McNally K.A., Lim W.A. (2016). Engineering T Cells with Customized Therapeutic Response Programs Using Synthetic Notch Receptors. Cell.

[103] Jaspers J.E., Brentjens R.J. (2017). Development of CAR T cells designed to improve antitumor efficacy and safety. Pharmacol. Ther..

[104] Srivastava S., Salter A.I., Liggitt D., Yechan-Gunja S., Sarvothama M., Cooper K., Smythe K.S., Dudakov J.A., Pierce R.H., Rader C. (2019). Logic-Gated ROR1 Chimeric Antigen Receptor Expression Rescues T Cell-Mediated Toxicity to Normal Tissues and Enables Selective Tumor Targeting. Cancer Cell.

[105] Roybal K.T., Rupp L.J., Morsut L., Walker W.J., McNally K.A., Park J.S., Lim W.A. (2016). Precision Tumor Recognition by T Cells with Combinatorial Antigen-Sensing Circuits. Cell.

[106] Sukumaran S., Watanabe N., Bajgain P., Raja K., Mohammed S., Fisher W.E., Brenner M.K., Leen A.M., Vera J.F. (2018). Enhancing the Potency and Specificity of Engineered T Cells for Cancer Treatment. Cancer Discov..

[107] Fedorov V.D., Themeli M., Sadelain M. (2013). PD-1– and CTLA-4–Based Inhibitory Chimeric Antigen Receptors (iCARs) Divert Off-Target Immunotherapy Responses. Sci. Transl. Med..

[108] Hegde M., Corder A., Chow K.K., Mukherjee M., Ashoori A., Kew Y., Zhang Y.J., Baskin D.S., Merchant F.A., Brawley V.S. (2013). Combinational Targeting Offsets Antigen Escape and Enhances Effector Functions of Adoptively Transferred T Cells in Glioblastoma. Mol. Ther..

[109] Ruella M., Barrett D.M., Kenderian S.S., Shestova O., Hofmann T.J., Perazzelli J., Klichinsky M., Aikawa V., Nazimuddin F., Kozlowski M. (2016). Dual CD19 and CD123 targeting prevents antigen-loss relapses after CD19-directed immunotherapies. J. Clin. Investig..

[110] Feng K., Guo Y., Liu Y., Dai H., Wang Y., Lv H., Huang J., Yang Q., Han W. (2017). Cocktail treatment with EGFR-specific and CD133-specific chimeric antigen receptor-modified T cells in a patient with advanced cholangiocarcinoma. J. Hematol. Oncol..

[111] Zah E., Lin M.-Y., Silva-Benedict A., Jensen M.C., Chen Y.Y. (2016). T cells expressing CD19/CD20 bi-specific chimeric antigen receptors prevent antigen escape by malignant B cells. Cancer Immunol. Res..

[112] Grada Z., Hegde M., Byrd T., Shaffer D.R., Ghazi A., Brawley V.S., Corder A., Schönfeld K., Koch J., Dotti G. (2013). TanCAR: A Novel Bispecific Chimeric Antigen Receptor for Cancer Immunotherapy. Mol. Ther. Nucleic Acids.

[113] Hegde M., Mukherjee M., Grada Z., Pignata A., Landi D., Navai S.A., Wakefield A., Fousek K., Bielamowicz K., Chow K.K.H. (2016). Tandem CAR T cells targeting HER2 and IL13Rα2 mitigate tumor antigen escape. J. Clin. Investig..

[114] De Munter S., Ingels J., Goetgeluk G., Bonte S., Pille M., Weening K., Kerre T., Abken H., Vandekerckhove B. (2018). Nanobody Based Dual Specific CARs. Int. J. Mol. Sci..

[115] Jia H., Wang Z., Wang Y., Liu Y., Dai H., Tong C., Guo Y., Guo B., Ti D., Han X. (2019). Haploidentical CD19/CD22 bispecific CAR-T cells induced MRD-negative remission in a patient with relapsed and refractory adult B-ALL after haploidentical hematopoietic stem cell transplantation. J. Hematol. Oncol..

[116] Bielamowicz K., Fousek K., Byrd T.T., Samaha H., Mukherjee M., Aware N., Wu M.-F., Orange J.S., Sumazin P., Man T.-K. (2018). Trivalent CAR T cells overcome interpatient antigenic variability in glioblastoma. Neuro Oncol..

